# A Treatise on Diseases of the Skin

**Published:** 1902-11

**Authors:** 


### BOOK REVIEWS.

A Treatise on Diseases of the Skin. For the use of Advanced Students and Practitioners. By Henry W. Stelwagon, M. D., Ph. D., Clinical Professor of Dermatology, Jefferson Medical College and Woman's Medical College, Philadelphia; Dermatologist to the Howard and Philadelphia Hospitals. Octavo, 1125 pages, with 220 text-illustrations, and 26 full-page lithographic and half-tone plates. Philadelphia and London: W. B. Saunders & Co. 1902. [Cloth, $6.00 net; Sheep or half-morocco, $7.00 net.]

Dr. Stelwagon's work is sure to win for itself a high place among the more pretentious textbooks relating to affections of the skin. The task of preparing this book has been unusually arduous, on account of the introduction of a vast amount of absolutely new material brought to light by more recent discoveries and developments. Of course, a considerable number of the subjects must necessarily have been derived from the foreign schools, but we are glad to notice that the merit of our American dermatological contributors has been specifically recognised in this volume.

It has been the habit of previous American authors to pass by the work of their own countrymen and substitute the reviews of foreign writers, which, in many instances, are inferior to those of our own; so that a work combining the two, and enriched by the criticisms originating in so keen a mind as that of Dr. Stelwagon, must be of the highest interest and the greatest possible value.

The classification adopted is that of Hebra, with modifications by Crocker and Morrow. It is, of course, still difficult to find any basis of classification that does not distinguish between affections which ought to be found in close juxtaposition. A classification constituted from an etiological point of view must of necessity be truest to nature, and is invaluable from a practical standpoint.

Hebra's classification is not adapted to the modern idea, as it embraces too many old traditions and often impedes more than it helps. Nothing seems possible, at present, but to postpone this whole matter to some future time when the bacteriology and pathology of the several dermatoses shall be better understood. Comparing a treatise of equal size published twenty years ago with the present one, it is easy to discover, not only a marked increase of diseases, but also a further separation of affections formerly included under a single designation.

It requires a clear and most intelligent judgment to sift from the profuse crop of dermatological literature that which is most likely to prove of value, and to assign it to its proper place and importance, in which attempt the author has perfectly succeeded. The references throughout the volume are conveniently arranged as foot-notes, and their value can hardly be over-estimated. We do not know of a similar English work where the references are so ample and admirable.

The introductory part of the book is on the anatomy and physiology of the skin. It is singularly short, although corresponding to the treatment of the subject as given by other authors. This might have been improved upon by presenting more correlations, with the purpose of leading up to the subsequent description of the pathological processes.

A number of new subjects are here treated, which have either come to light since the preparation of the last textbook, or have been crystalised from more or less vague conceptions into clearly drawn entities. Porakeratosis variegata, erythroderma pityriasique en plaques dissinnirees are placed under separate headings, although, according to the late conclusions of T. Colcott Fox and J. M. H. Macleod, these two conditions could be properly classed together. A papular persistent dermatosis, described by Johnston and yet unnamed, this author puts in juxtaposition with prurigo, which would more properly be included in the urticarial group.

Inasmuch as it would be impossible to discuss all the articles in a review of this kind, a slight reference will be made to the more common affections. In discussing eczema, the writer confines himself to the common phases of the disease. The multiplicity of exciting and predisposing causes is recognised, although the doctor's position regarding the parasitic theory is left to conjecture. The recent work of Bockhart (*Monatshefte für praktische Dermatologie*) upon this subject should have received consideration by the writer. His discussion of eczema is very much after the style of Duhring in his work on Cutaneous Medicine. Much space is devoted to diagnosis and the treatment of each variety of the disease, and it would seem that the best dermatologist could not fail to receive new hints concerning both.

The article upon impetigo contagioso is characterised by lack of recognition of the large increase of this disease among adults within the last few years and the omission of the fact that the disease so often asserts the ring,—a fact emphasised by the unnumbered epidemics incident to the last few years. The illustrating photographs are fairly good, lacking, however, sufficient explanation and comment.

A valuable factor of the work consists in combining into a single group the tuberculous diseases of the skin—papilloma, anatomic wart, lupus vulgaris and tuberculous cutis—all due to the bacillus of Koch. These were formerly classed under separate headings in different parts of the book, but are now found under the generic title of tuberculosis cutis. We commend Dr. Stelwagon for this sensible innovation, and wish he had gone even further and included the dermatoses of scrofulous subjects, so cleverly classified by Johnston under the name of paratuberculoses.

The defects in Dr. Stelwagon's book are hardly worth considering when contrasted with its merits. The work deals with what is practical in the science, together with the etiology and pathology. It is replete with information which has not merely been gathered but absorbed by the writer and assimilated with his almost unlimited experience. The clinical and pathologic features are emphasised by an extraordinary number of illustrations, the best of which are reproduced from Mracek's Hand Atlas, which contains the finest colored pictures yet given to the public. One must not, however, forget the value of black and white pictures, which when well done—as in this instance—are better than the average of the colored. Dr. Stelwagon has presented in this work a large number of illustrations from his own collection, with many reproduced from other authors—most of which are of genuine importance.

The subject of treatment is well handled. The usual multitudinous and often confusing references to various authorities are here wisely omitted. Dr. Stelwagon states clearly and concisely what a large experience has taught him, and outlines the treatment which has been found to be most efficient.

The book has been written for students and practitioners of medicine and fully accomplishes its purpose. On the whole, it is the most complete and satisfactory treatise on skin diseases in the English language. To the student it will prove of inestimable value on account of its clearness, conciseness and logical sequences, as well as its numerous apt illustrations; to the practitioner of medicine, it will be of great service—by means of it he will gain a more comprehensive insight into the particulars of one of the exact sciences and, at the same time, receive valuable information respecting other departments of medicine. To the expert dermatologist it will be of great interest, furnishing him with new hints and leading him into fresh avenues of thought. The mechanical portions of the book are worthy of all praise—clear text, striking captions, tasteful arrangement and neat and sensible binding. G. W. W.

